# Supplementary material for: Experimental realization of two-dimensional Dirac nodal line fermions in monolayer Cu2Si
Source: Nat Commun. 2017 Oct 18;8:1007. doi: 10.1038/s41467-017-01108-z (PMC5647340; doi:10.1038/s41467-017-01108-z)
Supplement: Supplementary file 1 — Supplementary Information [file 41467_2017_1108_MOESM1_ESM.pdf]

Supplementary Note 1: Orbital analysis

In Supplementary Figure 1, we show the orbital projected band structures of Cu_2Si. We find that the α and β bands are mainly composed of Si p_x/p_y, Cu d_{xy}, Cu $d_{x^2-y^2}$ and Cu p_x/p_y. All these orbitals are even with respect to the mirror operation M_z. The γ band is composed of mainly Si p_z, and partially Cu p_z, d_{xz} and d_{yz} orbitals, which are odd with respect to M_z. Thus, the Bloch waves composed of those orbitals are supposed to have definite eigenvalue of M_z. When two bands with opposite eigenvalues cross each other, such as α and γ bands, no gap will open at the degenerate points.

Supplementary Note 2: Symmetry protected Dirac points

In Fig. 2, we show that the breaking of mirror symmetry will lead to a band gap opening at the Dirac nodal lines. However, there are still gapless Dirac points along the ΓM and ΓK directions [Figs. 2(c) and 2(d)]. These gapless Dirac points are protected by other crystal symmetries.

We first consider the case in Fig. 2(a), *i.e.*, the neighbouring Cu atoms have an out-of-plane buckling with equal distance. The point group is D_{3d}. Along the ΓM direction in the BZ, the little group have out-of-plane mirror symmetry: M_{σ_1}, M_{σ_2}, and M_{σ_3}, as indicated by the red dashed lines in Supplementary Figure 2(a). The parity of M_σ for the three bands along the ΓM direction is shown in Supplementary Figure 2(c). The bands with the same parity will hybridize with each other and a gap will form at the crossing point. On the other hand, the bands with opposite parity remain gapless, forming the Dirac point. Along the ΓK direction, the little group have C_2 operation. The parity of C_2 is also shown in Supplementary Figure 2(a) which can nicely explain the remaining Dirac point. So our results indicate that the Dirac points along the ΓM and ΓK directions are protected by M_σ and C_2 symmetry, respectively.

We then consider the case in the Fig. 2(b), *i.e.*, the Si atoms are shifted in the out-of-plane direction. The point group is C_{6v}. A similar analysis reveals that the Dirac points along both the ΓM and ΓK directions are protected by the out-of-plane mirror symmetry: M_σ and M_τ, respectively.

Supplementary Note 3: Structure characterization

We prepared monolayer Cu_2Si by depositing Si onto a single-crystal Cu(111) surface. Si was evaporated from a Si(111) wafer by direct-current heating. The Cu(111) was cleaned by repeated sputtering and annealing circles. During growth, the substrate temperature was kept at approximately 500 K.

After preparation, we performed low-energy electron diffraction (LEED) and scanning tunneling microscopy to check the surface. From the large-scale STM image [Supplementary Figure 3(a)], we find that Cu_2Si can cover the whole terrace of Cu(111). We also note that there exist meandering strips that are randomly distributed on the surface. These stripes originate from the domain walls of different phases of Cu_2Si with Si atoms occupying FCC, HCP, and 2-fold

bridge sites [1,2]. From the high-resolution STM image in Supplementary Figure 3(b), we find that the lattice of Cu_2Si is continuous across the stripes, which shows the high quality of the sample. The lattice constant of Cu_2Si is approximately 4.3 Å, 30° from the [1-10] direction of Cu(111), which corresponds to a (√3×√3)R30° superstructure with respect to the Cu(111)-1×1 lattice. This (√3×√3)R30° superstructure has been directly observed in our LEED patterns, as shown in supplementary Figure 3(c).

To confirm the surface components of the Cu_2Si/Cu(111) sample, we performed high-resolution X-ray photoelectron spectroscopy measurements with 130-eV photons. Supplementary Figure 4(a) shows a survey of the sample, which contains Si 2p, Cu 3p, and Cu 3d peaks. In Supplementary Figures 4(b) and 4(c), we show the XPS data measured at two different emission angles (normal emission and grazing angle from the normal emission) for the Si 2p and Cu 3p peaks respectively. For the Si 2p peaks, the intensity is similar for the two geometries. This fact confirms that the Si atoms are located on the surface, which means that the Cu_2Si only form at the surface. On the other hand, we do find small shoulders for both $2p_{1/2}$ and $2p_{3/2}$ peaks (indicated by the black arrows), which is evidence for the existence of bulk-like silicon. The bulk-like silicon may originate from the small clusters on the surface, as shown in the STM images [Supplementary Figures 3(a) and 3(b)]. The Si clusters might play a role in the release of the strain because there is a slight lattice mismatch between Cu_2Si and the substrate.

For the Cu 3p peaks, the intensity measured with the grazing angle is much lower. This means that there are significant contributions from the bulk, *i.e.*, the Cu(111) substrate.

The ratio of Cu and Si atoms can be evaluated based on the area of the peaks. We use the grazing angle data to extract the information from the surface and find that Cu:Si~1.8:1. This means that there are excess Si atoms on the surface. The excess Si atoms may form additional clusters, as evidenced by the STM images [Supplementary Figures 3(a) and 3(b)].

Supplementary Note 4: Calculations with substrates

When Cu_2Si is grown on Cu(111), there are three possible phases [3], with Si atoms occupying the FCC, HCP and bridge sites [Supplementary Figures 5(a)-5(c)]. After structure optimization, the surface Cu_2Si layers in all the three phases remain relatively flat, with only negligible buckling (<0.07 Å). This result indicates that the mirror reflection symmetry M_z is preserved to a large extent, which is crucial for the survival of the nodal lines.

The calculated band structures of the three phases are shown in Supplementary Figures 5(d)-5(f), which are very similar to each other. We find that all the three bands (α, β and γ) are preserved. In particular, the intensity of the α and β bands are quite strong, with only a negligible gap when they cross the γ band. The intensity of the γ band is relatively weak because of the possible hybridization with the substrate. This result can explain our experimental observations that the γ band is much weaker than the α and β bands (Figs. 3 and 4). It should be noted that the γ band is mainly derived from the Si p_z, Cu p_z, d_{xz} and d_{yz} orbitals (Supplementary Figure 1). These orbitals have out-of-plane compositions, which prefer to hybridize with the Cu(111) substrate.

On the other hand, there are several γ-like bands in Supplementary Figures 5(d)-5(f), located at slightly higher and lower binding energies. To exclude the possibility of these bands, we calculated the band structures of the Cu_2Si layer and the substrate calculated separately, as shown

in Supplementary Figure 5(g), which supports our assignment of the γ band. Supplementary Figure 5(h) shows the bands of Cu_2Si/Cu(111) with artificially increased distance, which is expected to enhance the intensity of γ band. One can find that the intensity of the γ band becomes much stronger compared with the other similar bands. These results support our assignment of the γ band and indicate that the neighbouring bands originate from the Cu(111) substrate. However, only the γ band is visible in our experimental data. One possible reason is that the photon energy we used is in the ultraviolet regime, which is more sensitive to the surface bands.

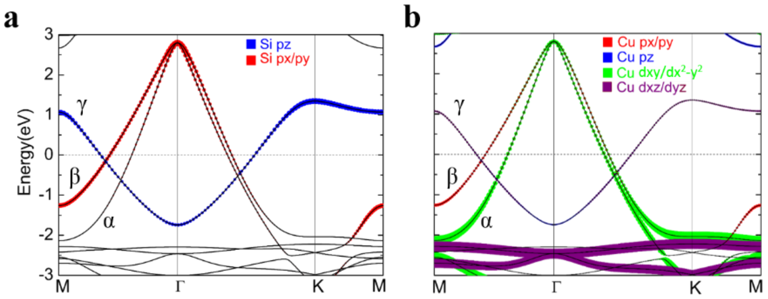

Supplementary Figure 1: Orbital projected band structures of free-standing Cu_2Si. (a) Si orbitals; (b) Cu orbitals.

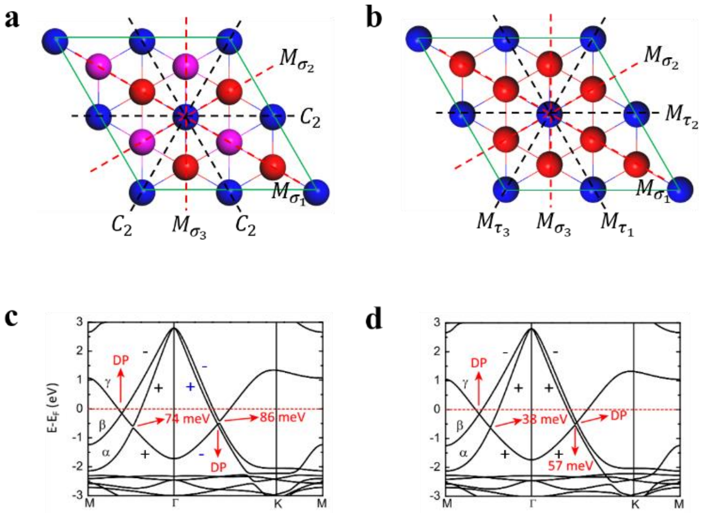

Supplementary Figure 2: Symmetry protected Dirac points. (a,b) Top view of the two configurations that break the mirror symmetry in Figs. 2(a) and 2(b). (a) The blue balls represent the Si atoms; the red and pink balls represent the Cu atoms that buckled upward and downward, respectively. (b) The blue balls represent the Si atoms; the red balls correspond to the coplanar Cu atoms. (c) and (d) Band structures of (a) and (b). The black and blue signs represent the parity of out-of-plane mirror symmetry and C_2 rotation symmetry, respectively.

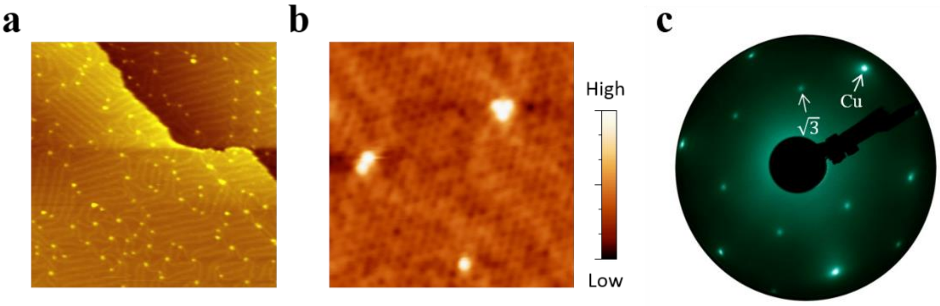

Supplementary Figure 3: Structure characterizations of monolayer Cu_2Si on Cu(111). (a,b) STM images of Cu_2Si on Cu(111). Size: (a) 80×80 nm^2; (b) 10×10 nm^2. (c) LEED pattern of Cu_2Si on Cu(111) measured using a beam energy of 65 eV.

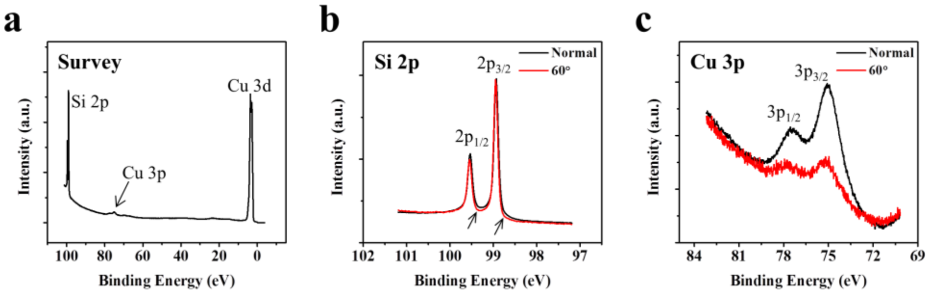

Supplementary Figure 4: X-ray photoelectron spectroscopy measurements. (a) Survey of Cu_2Si on Cu(111) measured with 130-eV photons. (b) XPS data measured on the Si 2p peaks with different configurations. Black line: normal emission; red line: 60° from the normal emission. (c) The same as (b) but for Cu 3p peaks.

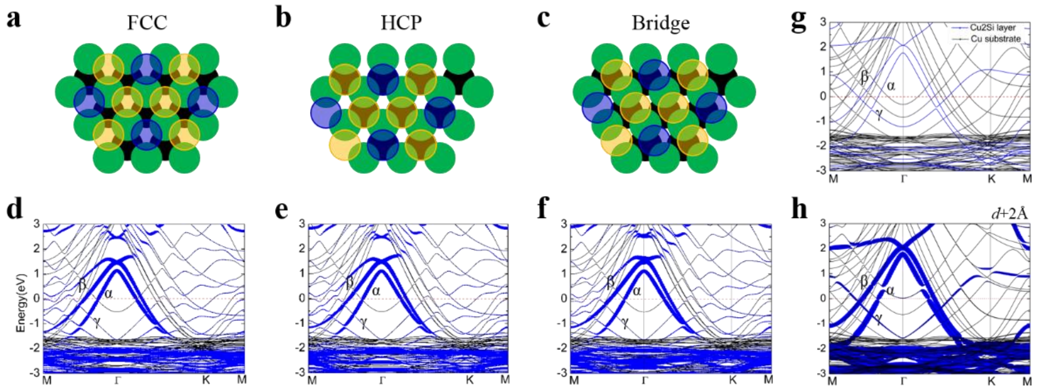

Supplementary Figure 5: First-principles calculation results including the substrate. (a-c) Three different phases of monolayer Cu_2Si on Cu(111), with Si atoms occupying the FCC, HCP, and bridge sites, respectively. (d-f) Calculated band structures according to the structural model in (a-c). Six layers of Cu atoms were considered in the calculations. The thickness of the lines illustrates the spectral weight. (g) Band structures of the Cu_2Si layer and the substrate calculated separately for the FCC phase. (h) Band structures of the FCC phase after increasing the distance of the Cu_2Si layer and the substrate by 2 Å.

Supplementary References:

[1] Shuttleworth, I. G., Fisher, C. J., Lee, J. J., Jones, R. G. & Woodruff, D. P. A NIXSW structural investigation of the (√3×√3)R30 º-Cu_2Si surface alloy phase formed by SiH_4 reaction with Cu(111). *Surf. Sci.* **491,** L645-L650 (2001).
[2] Shuttleworth, I. G. Deduction of a three-phase model for the (√3×√3)R30 º- Cu_2Si/Cu(111) surface alloy. *Appl. Surf. Sci.* **256,** 636-639 (2009).
[3] Shuttleworth, I.G. Investigation of the (√3 × √3)R30◦-Cu2Si/Cu(1 1 1) surface alloy using DFT. *Appl. Surf. Sci.* **257**, 6792-6798 (2011).
